# Quercetin-induced inhibition and synergistic activity with cisplatin – a chemotherapeutic strategy for nasopharyngeal carcinoma cells

**DOI:** 10.1186/1475-2867-12-34

**Published:** 2012-07-18

**Authors:** Maelinda Daker, Munirah Ahmad, Alan SB Khoo

**Affiliations:** 1 Molecular Pathology Unit, Institute for Medical Research, Jalan Pahang, Kuala Lumpur, 50588, Malaysia

**Keywords:** Cell proliferation, Chemotherapy, Cisplatin, Nasopharyngeal carcinoma, Quercetin, Synergism

## Abstract

**Background:**

Nasopharyngeal carcinoma (NPC) is a unique tumour of epithelial origin with a distinct geographical distribution, genetic predisposition and environmental as well as dietary influence as aetiological factors. Standard NPC treatment regimes, such as radiotherapy and concurrent chemotherapy with cytotoxic drugs, can produce undesirable complications often associated with significant toxicity. Here, we report the effects of a widely distributed flavonoid, quercetin, on cell proliferation, apoptosis and cell cycle arrest. The effects of combining quercetin and cisplatin on human NPC cells were explored.

**Methods:**

Cell proliferation was monitored by the dynamic, impedance-based cell analyzer (xCELLigence system) and the MTS assay. Ki67 proliferation antigen and fatty acid synthase (FASN) level was examined by Western blotting. Flow cytometry was also carried out to study the effects of quercetin on cell cycle and apoptosis status.

**Results:**

At 100 μM, quercetin inhibited cell proliferation and decreased expression of FASN and Ki67 antigen. Cell cycle analysis revealed a substantial increase in the proportion of cells in the G2/M phase. We also demonstrated the enhanced cytotoxic effects of quercetin treatment in concomitant with the chemotherapeutic drug, cisplatin, in cultured NPC cells. The combination index (CI) value of quercetin-cisplatin combination was < 1, indicating synergism.

**Conclusions:**

Our study showed that quercetin exhibited synergistic effects with cisplatin against NPC cells. Dose-reduction index (DRI) values > 1 implied the possibility of reducing the cisplatin dosage required to treat NPC, with the addition of quercetin. In turn, this could reduce the risk of cisplatin-associated toxicity. The potential of combining quercetin with cisplatin as a chemotherapeutic strategy for treatment of NPC should be explored further.

## Introduction

Nasopharyngeal carcinoma (NPC), a disease with distinct ethnic and geographic distribution, is common in Southern China, North Africa and South East Asia including Malaysia [1]. Approximately 95% of NPC cases [2] are strongly associated with latent infection of the Epstein-Barr virus (EBV).

Radiotherapy is the primary modality of treatment for NPC. In addition, concurrent chemotherapy with cisplatin and 5-fluorouracil is used. High survival rates are reported for stage 1 and stage 2 diseases [2]. Unfortunately, even with combined radiation and chemotherapy treatment, the rate of disease relapse is as high as 82% [2] and patients with locoregionally advanced disease, which most patients present, will eventually fail with distant metastases [3]. Improving outcomes are not without side-effects and are often linked to toxicity [4]. In addition, drug resistance may hamper the efficacy of these anticancer drugs. Potential NPC chemotherapy *via* natural product administration may circumvent some of these chemical- and radiation-associated toxicities.

Quercetin (3,3’,4’,5,7-pentahydroxyflavone) is a polyphenolic flavonoid widely distributed in fruits and vegetables, including apple, blueberries, broccoli, grape, leek, lettuce, onion and tomato. Being in a class of integral flavonoids common in our diet, it should exert low toxicity [5]. Quercetin may act on multiple targets. The mechanisms responsible for the cancer-preventive effects of quercetin and other flavonoids are attributed to their anti-oxidative activity, inhibition of enzymes that activate carcinogens, modification of signal transduction pathways, and interactions with receptors and/or proteins, described in [6]. Quercetin was reported to inhibit enzymatic activity of fatty acid synthase (FASN) and arrest cell growth [7]. It was shown to effectively inhibit growth in NPC HEN1 cells [8], human head and neck squamous cell carcinoma [9], human leukemic T-cells [10], human gastric cancer HGC-27 cells [11] and modulates Caco-2 human colorectal adenocarcinoma cell proliferation in a biphasic way [12]. To overcome obstacles associated with the limitation of cancer chemotherapy, possible biochemical modulation of anticancer drugs in concomitant with other agents, have been evaluated [13]. Here, we report the effects of quercetin treatment alone and in combination with cisplatin, in a set of EBV-negative and EBV-positive NPC cell line.

## Results

### Dynamic monitoring of quercetin-treated cells

**Figure 1:**
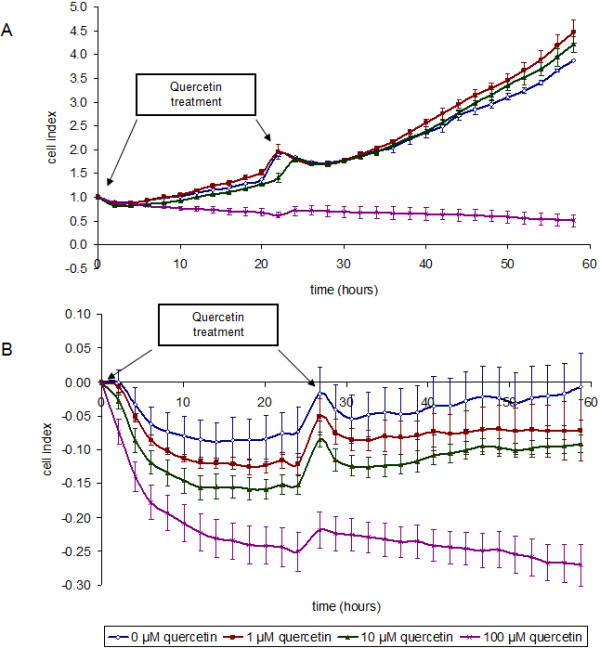
**Growth curves generated from acquired cell index depicting the effect of quercetin on (A) HK1 and (B) C666-1 cell proliferation.** 100 μM quercetin inhibited HK1 and C666-1 cell proliferation. The graph shown is a representative of triplicate experiments.

To determine the effect of quercetin on NPC cells, the xCELLigence system, a real-time cell proliferation, viability and cytotoxicity analyzer, was conducted. The assay involved exposing cells to 0 – 100 μM quercetin. The cell-electrode impedance response generated from the interaction of cells with the electronic biosensors was used to derive the cell index, representing growth over time (Figure 1a). At 100 μM, quercetin inhibited NPC cell proliferation. Essentially similar results were obtained when the experiment was repeated using C666-1 cells (Figure 1b), proving the consistent effect of quercetin in EBV-negative and EBV-positive NPC cells.

**Figure 2:**
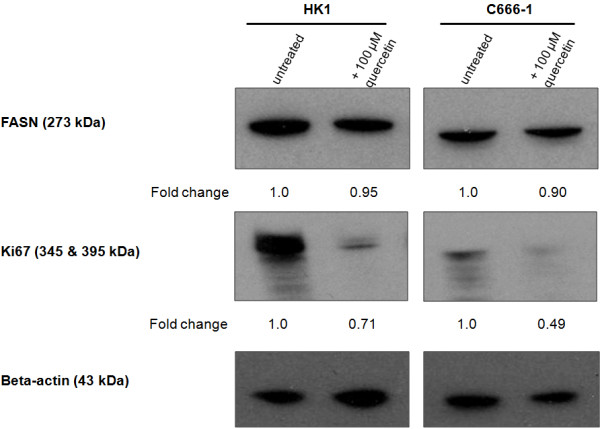
**Immunoblot for FASN and Ki67 24 hours after quercetin treatment.** HK1 and C666-1 cells treated with quercetin expressed lower amount of FASN and Ki67 compared to untreated cells. Detection of β-actin served as a loading control. The experiment was repeated twice and consistent results were obtained. A representative result is shown.

Because human cancer cells have elevated levels of fatty acid synthase (FASN) and undergo exacerbated endogenous fatty acid synthesis to maintain a constant supply of lipids and lipids precursors for membrane production in a highly-proliferating population [14] and the Ki67 antigen is a cell proliferation marker, quantification of FASN and Ki67 in HK1 cells was performed. Since there is no standard method for interpreting Ki67 immunostaining (for example, area of view selected for scoring and/or number of cells for scoring) and various methods may account for the diverse results reported, we attempted to quantify, *via* Western blotting, FASN and Ki67 antigen expression to assess their link to proliferative activity. We showed that FASN expression was marginally lowered by quercetin (Figure 2) following quercetin-induced cell proliferation inhibition. Simultaneously, the downregulation of Ki-67 expression was clearly observed (Figure 2). The C666-1 cell line was included for comparison. This was in agreement with Figure 1 which revealed that proliferation was abrogated in the presence of 100 μM quercetin. Taken together, similar results proved the consistent effect of quercetin in NPC cells.

### Minor contribution of quercetin to apoptosis

**Figure 3:**
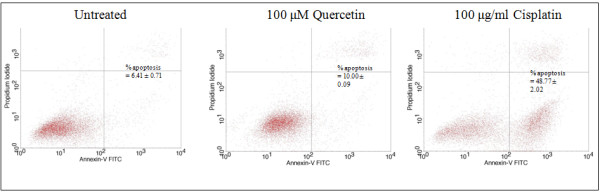
**Flow cytometry analysis using annexin V-FITC/PI double staining depicting apoptosis.** The lower right and upper right quadrants represent cells undergoing apoptosis. Images shown are representatives of three independent experiments. The average percentage ± sd of total apoptotic cells is shown.

To determine if the quercetin-induced static impedance registered at 100 μM was associated with apoptosis, HK1 cells exposed or not exposed to quercetin were incubated with FITC-conjugated annexin V and propidium iodide. Apoptotic cells were identified by flow cytometry (Figure 3). Treatment with 100 μM quercetin for one day did not contribute markedly to cancer cell death. Hence, apoptosis is not the main mechanism of quercetin-induced growth inhibition. A comparison with cisplatin, a drug currently in use for NPC chemotherapy, is shown. Cisplatin clearly induced apoptosis in HK1 cells.

### Cell cycle arrest by quercetin

**Figure 4:**
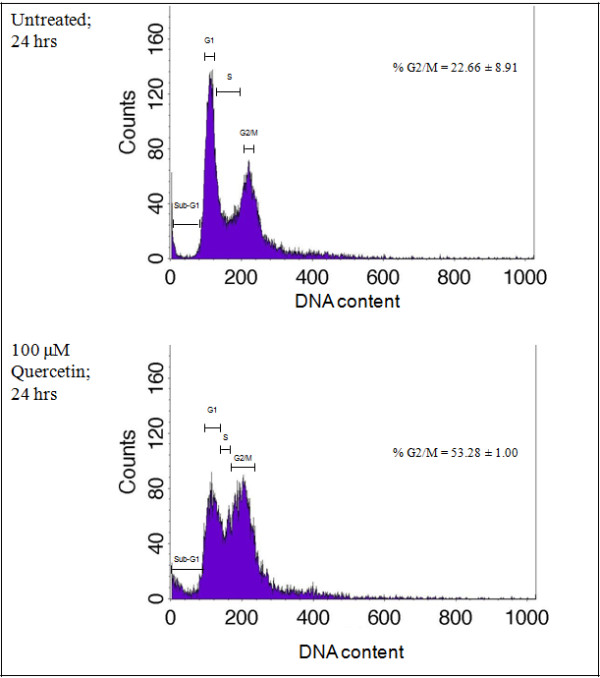
**Cell cycle analysis by BrdU labelling in untreated and quercetin-treated cells.** Increasing percentages of cells in G2/M phase and sub-G_1_ peak were observed. A representative of three independent experiments is shown. The average percentage ± sd of total cells in the G2/M phase is shown.

To find out if exposure to quercetin affects cell cycle, DNA content was determined by flow cytometry. After 24 hours of 100 μM quercetin exposure, there was a substantial increase of cells in G2/M phase in HK1 cells compared to untreated cells (Figure 4).

### Quercetin inhibits proliferation alone and displayed synergistic effects in combination with cisplatin

**Figure 5:**
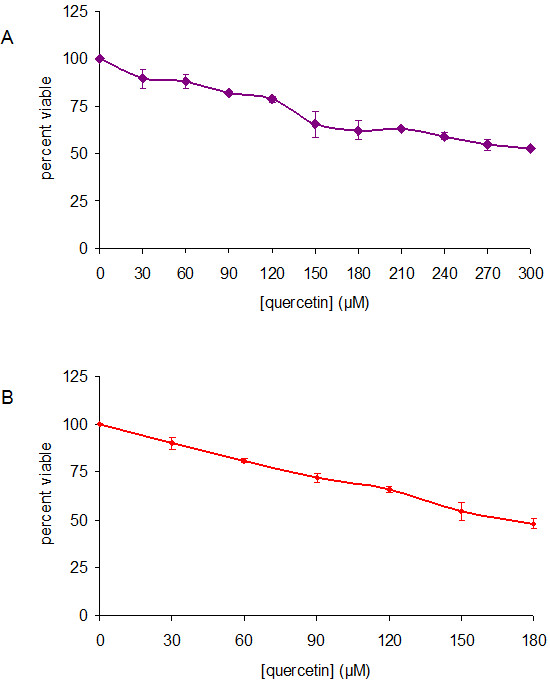
**Dose–response curves of quercetin-treated HK1 (A) and C666-1 (B) cells.** The average percentage viable ± sd relative to vehicle control cells is shown.

**Figure 6:**
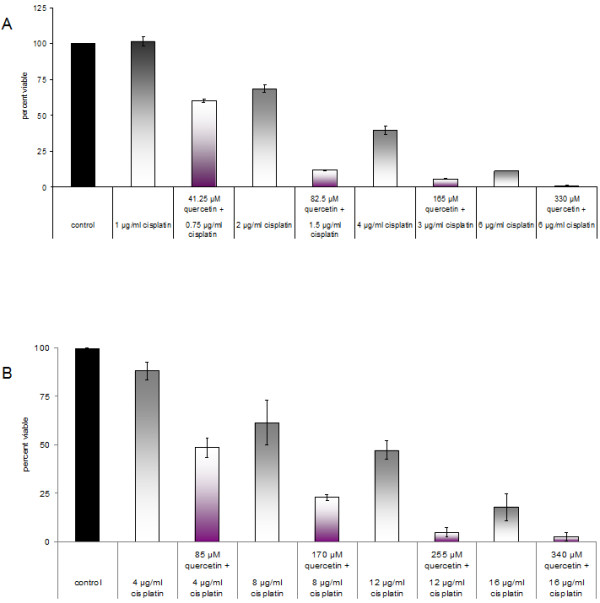
**Bar graphs showing viability after cisplatin or simultaneous treatment with cisplatin and quercetin in HK1 (A) and C666-1 (B) cells, determined by the MTS assay.** The control group on the extreme left is assigned 100% viability. The combination of quercetin and cisplatin is more active than cisplatin alone. Results are presented as average percentage viable ± sd.

**Figure 7:**
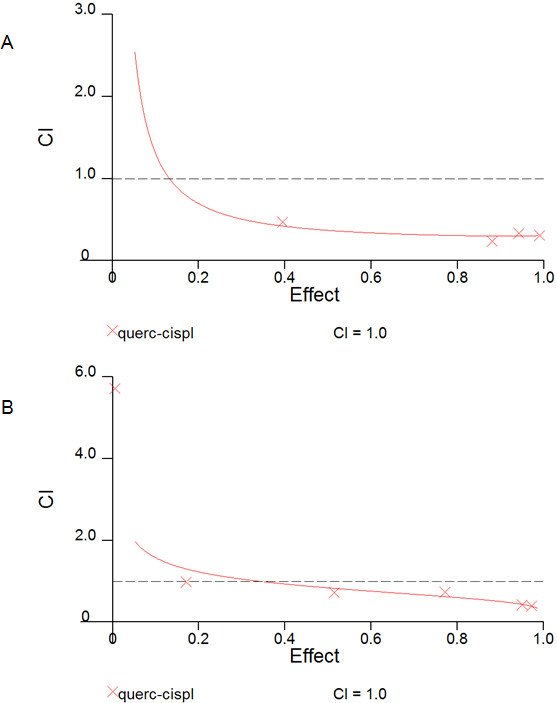
**F**_**a**_**-CI plots for HK1 (A) and C666-1 (B), respectively, showed a synergistic cytotoxic effect for simultaneous treatment of a fixed combination ratio of quercetin and cisplatin.** In the F_a_-CI plot, the dashed line (CI = 1) indicates an additive reaction between the two substances. Values above and below this dashed line imply antagonism and synergism, respectively.

Prior to assessing a two-drug combination effect on NPC cells, quercetin and cisplatin dose–response curves were generated for HK1 and C666-1. Cells in the solvent control group received DMSO only at the same concentration and volume as quercetin treatment and showed no influence on cell viability. Quercetin and cisplatin exerted their effects in a dose-dependent manner (Figure 5; cisplatin curves not shown). When NPC cells were grown in the presence or absence of quercetin and cisplatin, it was obvious that simultaneous treatment of a fixed combination ratio of quercetin and cisplatin exerted an effect greater than cisplatin alone (Figure 6). Using the CalcuSyn software, we determined the combination index (CI) to ascertain synergism (CI < 1), antagonism (CI > 1) or additive effect (CI = 1). The CI values are tabulated (Table 1). The CI method, described in [15], revealed a synergistic cytotoxic effect in NPC cells (Figure 7).

**Table 1:** Description of CI values for each fraction of cells affected and the corresponding dose reduction index

**NPC cell line**	**Fraction affected**	**CI**	**Description**	**Dose reduction index (DRI)**	
				**Quercetin**	**Cisplatin**
HK1	0.395	0.474	Synergism	6.142	3.209
	0.881	0.239	Strong synergism	128.972	4.319
	0.943	0.338	Synergism	222.314	2.998
	0.991	0.308	Synergism	2055.870	3.247
C666-1	0.171	0.981	Nearly additive	1.936	2.153
	0.515	0.718	Moderate synergism	3.531	2.299
	0.772	0.735	Moderate synergism	4.412	1.967
	0.951	0.425	Synergism	11.690	2.944
	0.974	0.402	Synergism	14.736	2.994

DRI values > 1 are beneficial and the greater the DRI values, the greater the dose reduction for a given therapeutic effect [15].

## Discussion

Many studies had reported the potential of the polyphenolic flavonoid, quercetin, as an anti-cancer agent. The interest in quercetin for therapeutics application is spurred by its ability to sensitize cells to the cytotoxic effects of platinum-containing drugs [16]. However, limited research is available on the role of quercetin in NPC and potentiation is seldom defined as additive or synergistic. Our work demonstrated that quercetin inhibited proliferation of NPC cells. Our data showed that quercetin synergizes with cisplatin in EBV-negative and EBV-positive NPC cells.

The limitation of cancer chemotherapy is cytotoxicity towards normal tissues and drug resistance. Intrinsic or acquired multidrug resistance (MDR) is a major impediment to successful chemotherapy. Quercetin showed growth inhibitory activity on both drug sensitive and MDR cells. Various findings with respect to modulating cancer MDR with quercetin had been comprehensively reviewed in [5]. The authors listed a number of studies demonstrating that natural products from plants such as flavonoids are potential drugs to overcome MDR in many multidrug resistant cells. They also summarized a number of literatures on the effect of quercetin on compounds of which cellular accumulation, transport or bioavailability was increased in different model systems. In addition, quercetin at a non-cytotoxic concentration has enhanced the effect of chemotherapeutic drug on MDR cells. The candidacy of quercetin as a chemosensitizer for the ABC pump-proteins has been tested in a number of MDR tumour cell lines [5].

The richest sources of quercetin were onions (347 mg/kg), apples (36 mg/kg) and red wine (11 mg/kg)[17]. The average intake of quercetin in the Netherlands was 16 mg/day, with the main sources being tea, onions and apples [18]. The estimated daily dietary intake of quercetin in the U.S., Europe and Asia was 4 – 68 mg [5]. In ref. [19], it was stated that after the consumption of 1 L red wine, 1 kg broccoli, 1 kg onions or 1 kg apples, the estimated doses quercetin that will be reached are 19, 30, 140 or 347 mg, respectively. From refs. [18, 20], by extrapolation, a serum concentration of 10 μM might be attained from a daily dose of 1500 mg. Since this concentration required for anti-cancer activity (>10 μM) is much higher than can be achieved by nutrition alone, a better source of quercetin are commercially available dietary quercetin tablets [19]. Repeated dietary intake of quercetin-containing foods would lead to accumulation of quercetin in the plasma [21]. Various studies on the pharmacokinetics analysis of quercetin in human plasma are available [22-24].

Cisplatin, often hampered by renal toxicity, preferentially accumulates in the cells of the proximal tubule. It was initially thought that a combination of quercetin and cisplatin might harm renal tubular cells and increased nephrotoxicity would limit the clinical value of a cisplatin-quercetin combination. It is of particular importance that in [25], quercetin reduced cisplatin-induced nephrotoxicity in porcine LLC-PK_1_ renal tubular epithelial cells. This is noteworthy of mention because *in vitro*[9,26,27] and *in vivo*[13], quercetin was shown to enhance the anti-proliferative effects of cisplatin. Given that quercetin and cisplatin exhibited synergism, this combination could be a potential therapeutic application of clinical value in the management and treatment of human NPC. The beneficial DRI values listed in Table 1 indicate that quercetin may make it possible to lower cisplatin doses to below toxic levels, thereby possibly reducing toxicity whilst maintaining or improving efficacy. Quercetin, by itself (Figures 3 and 5), appears to confer little toxicity, in agreement with [13] which stated that quercetin is a weakly toxic drug, and seemed more likely to confer cytostatic effects (Figure 4).

## Conclusion

In our study, quercetin consistently inhibited proliferation of NPC cells. The effect of quercetin was associated with cell cycle arrest in the G2/M phase. In addition, quercetin synergized with cisplatin in causing anti-proliferative effects in NPC cells. This study suggests that the potential of quercetin as a chemotherapeutic agent in human NPC should be explored further.

## Methods

### Cell lines and culture

HK1, an EBV-negative NPC cell line acquired from George Tsao and co-workers, was maintained in the exponential growth phase in RPMI 1640 medium (GIBCO, Invitrogen, Carlsbad, CA) supplemented with 10% heat-inactivated foetal calf serum (FCS; GIBCO), 10 U of penicillin per ml (GIBCO), and 10 μg streptomycin per ml (GIBCO) at 37 °C in a 5% CO_2_ humidified atmosphere. C666-1, an EBV-positive NPC cell line supplied by Kwok-Wai Lo, was maintained similarly but FCS concentration was increased to 15%. The establishment and characterization of HK1 was described in [28]; whereas that of C666-1 in [29]. The identity of HK1 and C666-1 were validated by DNA fingerprinting using AmpFISTR Identifiler® PCR amplification kit (Applied Biosystems, Foster City, CA). The short tandem repeat profiles were consistent with the original NPC cells [30]. Tests for detection of mycoplasma using *e*-myco™ Mycoplasma PCR Detection Kit (iNtRON Biotechnology, INC., Korea) were conducted routinely and contamination-free cells were used throughout this study.

### xCELLigence cell proliferation assay

HK1 cells were seeded at a density of 5000 cells/well into three E-Plate 16 (ACEA Biosciences, Inc., San Diego, CA) containing 100 μl medium per well supplemented with 10% FCS. When the cells have entered logarithmic phase, 1 mM quercetin (C_15_H_10_O_7_⋅xH_2_O, molecular weight = 302.24 anhydrous basis, purity ≥ 95%) from Sigma dissolved in dimethyl sulfoxide (DMSO) (Sigma) was prepared. Old culture medium was aspirated and replaced with culture medium containing quercetin to yield a final concentration of 1 – 100 μM. Final DMSO concentration in the cell culture did not exceed 0.5%. Cells were monitored for approximately 60 hours at 37 °C in a 5% CO_2_ atmosphere, with one change of freshly-prepared medium and quercetin at the appropriate concentrations at 24 hours post-treatment. Dynamic monitoring of the growth inhibition pattern was determined by the impedance-based xCELLigence system (Roche Applied Science, Germany). The cell index was derived from measured cell-electrode impedance that correlates with number of cells, viability, and/or cytotoxicity. Vehicle control cultures received DMSO alone. For C666-1, 15 000 cells/well were seeded and medium was supplemented with 15% FCS.

### Western blotting

1.2 x 10^6^ cells were seeded in 10-cm culture dishes and allowed to enter logarithmic phase prior to treatment, after which the cells were cultured for an additional 24 hours in medium without or with 100 μM quercetin. Cells were lysed in 1X RIPA lysis buffer and then boiled for 10 minutes. The quantity of protein in the cell lysate was determined by protein assay (Bio-Rad Laboratory, Hercules, CA). 10 μg of protein was resolved in NuPAGE® Novex® Bis-Tris Mini Gels (Invitrogen) and electrotransferred to polyvinylidene fluoride membranes (Millipore, Bedford, MA). The membranes were blocked with 5% skimmed milk and incubated overnight in primary antibodies diluted in 5% skimmed milk. Primary antibodies used include anti-FASN (Abcam, Cambridge, UK), anti-beta-actin (Santa Cruz Biotechnology, Inc., CA, USA) and monoclonal mouse anti-human Ki67 antigen (DakoCytomation Glostrup, Denmark). The secondary antibody reaction was carried out with anti-mouse or anti-rabbit horseradish peroxidase-conjugated IgG (Promega). Western Lighting® Plus ECL substrate (PerkinElmer, Waltham, MA) and autoradiography were employed for visualization of protein expression. Densitometry analysis of X-ray films was performed on Alpha Imager System (Alpha Innotech/ProteinSimple, Santa Clara, CA, USA) using Alpha View software.

### Apoptosis analysis assay

1.2 x 10^6^ HK1 cells were seeded in 10-cm culture dishes and were allowed to adhere overnight. Following this, cells were treated either with 100 μM quercetin, 100 μg/ml cisplatin, or DMSO vehicle (as control). All culture dishes were re-incubated for another 24 hours. Apoptosis was determined on a FACSCalibur flow cytometer (BD Biosciences, San Jose, CA), using the BD Pharmingen FITC Annexin V apoptosis detection kit (San Diego, CA), according to the manufacturer’s protocol provided.

### Cell cycle analysis assay

1.2 x 10^6^ HK1 cells were seeded in 10-cm culture dishes and left overnight; after which treated cells received 100 μM of quercetin for 24 hours whereas untreated control cultures received DMSO alone. Cultured cells were labelled with 10 μM BrdU in medium for 17 hours before they were harvested. Cell cycle distribution was determined on a FACSCalibur flow cytometer, using the BrdU incorporation method with the BD Pharmingen FITC BrdU Flow Kit (San Diego, CA).

### MTS [3-(4,5-dimethylthiazol-2-yl)-5-(3-carboxymethoxyphenyl)-2-(4-sulfophenyl)-2 H-tetrazolium] assay

5000 HK1 or 15 000 C666-1 cells/well were seeded into 96-well microtiter plates then incubated until logarithmic phase was achieved prior to treatment. Then, old medium was aspirated and the cells were incubated in 100 μl medium containing various concentrations of quercetin or cisplatin for 3 days at 37 °C in a 5% CO_2_ atmosphere. The number of viable cells at the end of the incubation period was measured using the CellTiter 96® AQ_ueous_ One Solution Cell Proliferation (MTS) assay (Promega, Madison, WI), according to the protocol provided by the manufacturer. Absorbance at 490 nm was read using the EnVision multilabel plate reader (PerkinElmer, Waltham, MA). Non-specific absorbance was measured at 630 nm. Wells containing the appropriate medium but without cells served as blank. Cell viability was calculated as percentage compared to control cells, which were arbitrarily assigned 100% viable and the growth curve was plotted. All experiments were performed in triplicates and values were reported as mean ± sd.

For combined drug analysis, a fixed ratio combination of quercetin and cisplatin was evaluated. The doses of quercetin and cisplatin were chosen based on IC_50_ values, defined as the concentration that inhibited 50% cell growth relative to control cells. For drug combination studies, plates were assigned for single-drug only and two-drug and the experiments were carried out simultaneously to rule out different experimental conditions [15]. Following drug addition, the 96-well microtiter plates were incubated for 3 days and the MTS assay was carried out to determine percent viability.

### Combined drug analysis

Drug interaction was determined by the combination-index (CI) method described in [15]. Dose–response curves, dose-effect analysis and CI for the combination treatment group were generated using CalcuSyn 2 software (Biosoft, Cambridge, UK). A CI of > 1 implies antagonism, CI = 1 is additivity and CI < 1 is synergy.

## Abbreviations

BrdU, Bromodeoxyuridine; CI, Combination index; DMSO, Dimethyl sulfoxide; DNA, Deoxyribonucleic acid; DRI, Dose-reduction index; EBV, Epstein-Barr virus; ECL, Enhanced chemiluminescence; FASN, Fatty acid synthase; FCS, Foetal calf serum; MDR, Multidrug resistance; MTS, [3-(4,5-dimethylthiazol-2-yl)-5-(3-carboxymethoxyphenyl)-2-(4-sulfophenyl)-2 H-tetrazolium]; NPC, Nasopharyngeal carcinoma; PCR, Polymerase chain reaction.

## Competing interests

The authors declare that they have no competing interests.

## Authors’ contributions

Conceived and designed the experiments: MD, Acquisition of data: MD, Analyzed and interpreted the data: MD, ASBK, MA. Wrote the paper: MD, Critical and intellectual revision of the article contents: ASBK, MA. All authors read and approved the final manuscript.
